# Standardization of A Physiologic Hypoparathyroidism Animal Model

**DOI:** 10.1371/journal.pone.0163911

**Published:** 2016-10-03

**Authors:** Soo Yeon Jung, Ha Yeong Kim, Hae Sang Park, Xiang Yun Yin, Sung Min Chung, Han Su Kim

**Affiliations:** 1 Department of Otorhinolaryngology-Head and Neck Surgery, School of Medicine, Ewha Womans University, Seoul, Korea; 2 Department of Molecular Medicine, School of Medicine, Ewha Womans University, Seoul, Korea; 3 Department of Otorhinolaryngology-Head and Neck Surgery, Chuncheon Sacred Heart Hospital, Hallym University, College of Medicine, Chuncheon, Korea; 4 Department Orthopaedic Surgery, School of Medicine, Ajou University, Suwon, Korea; Nanjing Medical University, CHINA

## Abstract

Ideal hypoparathyroidism animal models are a prerequisite to developing new treatment modalities for this disorder. The purpose of this study was to evaluate the feasibility of a model whereby rats were parathyroidectomized (PTX) using a fluorescent-identification method and the ideal calcium content of the diet was determined. Thirty male rats were divided into surgical sham (SHAM, n = 5) and PTX plus 0, 0.5, and 2% calcium diet groups (PTX-FC (n = 5), PTX-NC (n = 10), and PTX-HC (n = 10), respectively). Serum parathyroid hormone levels decreased to non-detectable levels in all PTX groups. All animals in the PTX—FC group died within 4 days after the operation. All animals survived when supplied calcium in the diet. However, serum calcium levels were higher in the PTX-HC than the SHAM group. The PTX-NC group demonstrated the most representative modeling of primary hypothyroidism. Serum calcium levels decreased and phosphorus levels increased, and bone volume was increased. All animals survived without further treatment and did not show nephrotoxicity including calcium deposits. These findings demonstrate that PTX animal models produced by using the fluorescent-identification method, and fed a 0.5% calcium diet, are appropriate for hypoparathyroidism treatment studies.

## Introduction

Hypoparathyroidism is a state of parathyroid hormone (PTH) deficiency that is induced by many medical conditions. The most common etiology is accidental excision of the parathyroid gland during thyroid surgery. [1, 2] PTH increases phosphorus excretion by the kidney, stimulates calcium absorption by the renal tubules and small intestine, and activates osteoclasts to enhance bone turnover. PTH also activates vitamin D in the kidney. PTH deficiency leads to a loss of calcium homeostasis and hypocalcemia. This condition causes neurophysiologic disturbances including paresthesia, weakness, irritability, and muscle cramping. [2]

Conventional treatment of hypoparathyroidism involves supplementation with calcium and vitamin D analogues. Patients must take the oral medications (2–8 tablets) daily. However, while calcium levels increase, they are not regulated physiologically by the medications. Bone metabolism and kidney function are also not regulated physiologically. Therefore, the incidence of complications, such as bone fractures and nephrolithiasis, are increased. [3] These limitations increase the demand for novel treatment modalities. Clinically, injection with recombinant human PTH (1–84) has been introduced. However, daily administration of PTH differs from normal responses to physiological demands. Experimentally, tissue-engineered parathyroid gland tissue has been studied. [4, 5] Auto-regulated and calcium-responsive parathyroid tissue would be the ideal treatment modality.

Reproducible animal models that fully mimic hypoparathyroidism pathophysiology are mandatory for evaluating the feasibility of new treatment modalities. Ideal animal models enable experiments that not only generate reliable research results, but also avoid ethical issues and safety problems. However, there are currently no representative hypoparathyroidism animal models.

Animal models of hypoparathyroidism have been reported previously by using surgical excision with a microscope and cauterization, and by visualization. [6–10] However, because the rat parathyroid is very small, it is difficult to identify and precisely excise this gland alone. Therefore, postoperative PTH levels are inconsistent. [9] We reported previously a surgically excised hypoparathyroidism animal model using the 5-animolevulinic acid (5-ALA) fluorescent-identification method. By using fluorescence detection, parathyroid glands were excised precisely without any remnant parathyroid tissue. This surgical technique successfully produced a hypoparathyroidism animal model that was documented by post-operative PTH decreasing rapidly to an undetectable level. [5, 11]

Ideal disease-specific animal models should reproduce the pathophysiology of the disease. For human primary hypoparathyroidism, this means that the animal model should demonstrate not only low PTH levels, but also comparable changes in the calcium and phosphorus balance. In our initial model, rats survived for the observational periods with no signs of hypocalcemia without calcium replacement, despite a low PTH level. [11] There was discrepancy with human hypoparathyroidism, because, in human, low serum calcium levels lead to death with undetectably low PTH levels without additional calcium supplement. Therefore, the diet formula was modified in the next study. A calcium-free diet (CFD) was supplied to all animals to avoid the effect of dietary calcium supplementation. [5] All animals died 2~3 days after the surgery with symptoms of severe hypocalcemia. However, when calcium was supplied to the animals, serum calcium levels were elevated and the animals survived. This model successfully mimicked the pathophysiology of primary hypoparathyroidism. However, the CFD is more expensive than normal commercial diets and the death of control animals was wasteful and costly.

The ideal diet formulation should be determined and evaluated considering both the physiological and financial aspects of the hypothyroidism animal model. The American Institute of Nutrition (AIN) has published information about purified diets for experimental rodents. [12] Among these diets, AIN-93G uses a mineral mix that strictly balances the calcium-phosphorus ratio. [13] In the current study, we determined the optimal dietary calcium content by using a CFD, AIN-93G, and a calcium-added diet, and evaluated the feasibility of our animal model by examining the pathophysiology of hypoparathyroidism.

## Material and Methods

### Animals

Thirty male Sprague-Dawley rats (Orient Bio, Sungnam, Korea), approximately 8 weeks of age and weighing 260–350 g, were used. All animals were acclimated for at least 7 days before the experiments. All animals were housed under a 12 h light/dark cycle and allowed free access to food and water. During the acclimation period, the same rodent chow was supplied for all the animals. Animal care followed the Guide for the Care and Use of Laboratory Animals by the Institute of Laboratory Animal Resources and the National Institutes of Health, and the Animal Experiment Guidelines of Ewha Womans University Medical Research Institute. This study was approved by the Committee for Ethics in Animal Experiments, Ewha Womans University Medical Research Institute.

### Design of the animal study

Animals were divided into following four groups according to the surgical procedure and calcium concentration of the diet.

1. Surgical sham (SHAM, exposing the parathyroid gland and closing the wound, n = 5).
2. Parathyroidectomized with calcium-free diet (PTX-FC, n = 5).
3. Parathyroidectomized with normal calcium diet (PTX-NC, n = 10).
4. Parathyroidectomized with high calcium diet (PTX-HC, n = 10).

AIN-93G (Research Diets, New Brunswick, NJ, USA) that contained 5 g/kg calcium (0.5%) was used as the normal diet for the SHAM and PTX-NC groups. Based on the AIN-93G formula, calcium-free (0%) and calcium-added (2%) formulas were produced as customized diets. Calcium and phosphorus contents are listed in Table 1 including the rodent chow (Cargill Agri Purina, Pyongtaek, Korea) that was used in former study. [11] The other ingredients of the diet formulations are listed in (S1 and S2 Tables). Diet consumption and weight changes were evaluated weekly.

**Table 1:** Calcium and phosphorus components of the diets. Abbreviations: Ca, calcium; P, phosphorus.

	AIN-93G	Calcium-added diet	Calcium-free diet	Rodent chow
	PTX NC, SHAM	PTX-HC	PTX-FC	
Ca (g/kg)	5	20	0	11.4
P (g/kg)	1.56	6.24	1.56	6.1
Ca: P ratio	3.20: 1	3.20: 1		1.86: 1

### Surgical excision of rat parathyroid glands

Parathyroid glands in all PTX groups were identified and removed according to the 5-ALA fluorescent-identification method. [11] Briefly, 5-ALA powder (Sigma-Aldrich Korea, Yongin, Korea) was suspended in a 0.5 mL of 0.9% sodium chloride solution. The resultant 5-ALA solution was administered (500 mg/kg) by intraperitoneal injection to the PTX and SHAM groups. All animals were kept under subdued light for 2 hours to prevent phototoxicity. After 2 hours, animals were anesthetized by intraperitoneal injection of Zoletile (Virbac Korea, Seoul, Korea) and xylazine chloride (Bayer Korea, Seoul, Korea) (1:1 mix, 0.1 mL/100 g). A vertical skin incision was made at the midline of the neck, and muscles were dissected until the trachea and thyroid gland were exposed. The red fluorescent parathyroid glands were detected under illumination of a xenon light (380–440 nm) source with an ultraviolet filter that was designed to detect fluorescence emission at 635 nm. Both parathyroid glands were removed in the PTX groups using a cold knife while the glands were retained in the SHAM group. Hemostasis was performed by gauze compression and bipolar cauterization. The skin incision was sutured with non-absorbable 4–0 Ethilon^®^ (Johnson & Johnson, New Brunswick, NJ, USA).

### Laboratory evaluation

PTH serum levels were measured on postoperative day 7 by an enzyme-linked immunosorbent assay (ELISA) (Rat Bioactive Intact PTH ELISA kit, Immutopics, San Clemente, CA, USA) to confirm the complete removal of parathyroid glands. The levels of serum calcium, serum phosphate, blood urea nitrogen (BUN), and serum creatinine (Cr) were evaluated with an automatic chemistry analyzer. Urine calcium and phosphorus levels were also measured. Serum osteocalcin and C-telopeptide of type-I collagen (CTX-1) levels were measured by rat osteocalcin (Immutopics) and rat C-telopeptide of type-I collagen ELISA kits (Cusabio, Wuhan, China), respectively. All parameters were evaluated pre-operatively, and 4 and 8 weeks post-operatively.

### Histological evaluation

Animals were sacrificed 8 weeks after the surgical procedure. Kidneys were obtained to evaluate the occurrence of nephrotoxicity. The specimens were embedded in paraffin blocks and stained with hematoxylin and eosin. Signs of kidney toxicity including tubular atrophy, interstitial fibrosis, interstitial inflammation, and glomeruli deformity were assessed. The presence of calcium deposit was also evaluated. The severity was graded as 0 = normal histology, 1 = < 25%, 2 = > 25%, but <50%, 3 = > 50%, but <75%, 4 = >75%. These finidngs were evaluated by a pathologist under the light microscope; four different fields of slide under × 100 magnification.

### Microcomputerized tomography (μCT)

**Fig 1:**
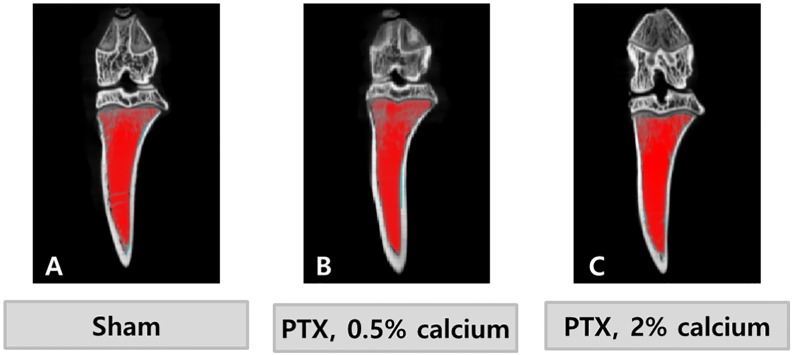
The region of interest (ROI) on microcomputerized tomography. A coronal view of the rat tibia. The ROI is indicated as red color.

Eight weeks after surgery, μCT (NFR Polaris-G90; NanoFocusRay, Jeonju, Korea) was performed on proximal tibia of nine animals (three animals from each of the SHAM, PTX-NC, and PTX-HC groups). The μCT settings were: 70kVP, 50 μA, 360 views, 500 scan numbers, and 512 × 512 reconstruction matrix. After 3-dimentional reconstruction, the bone volume fraction, trabecular thickness, trabecular number, trabecular separation, and connectivity density (Conn.D) were obtained at region of interest (Fig 1) by using Skyscan 1076 in vivo μCT scanner software (Skyscan, Aartselaar, Belgium).

### Statistical analyses

All statistical analyses were performed using SPSS (version19) (IBM, Chicago, IL, USA). Results are expressed as means ± standard deviations. Repeated measure analysis of variance was used to determine the statistical significance of weight changes between groups. The Kruskal-Wallis test was used to compare the results among the three groups and the Mann-Whitney test was used to determine statistical significance between two of the three groups. A *p* value < 0.05 was considered significant.

## Results

### Development of the hypoparathyroidism animal models

**Fig 2:**
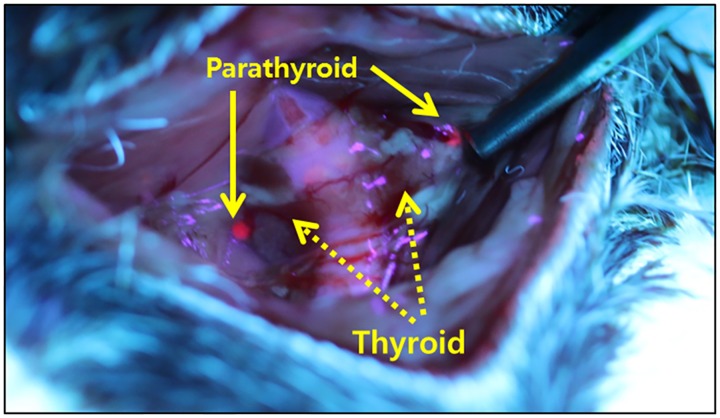
Identification of rat parathyroid glands. The parathyroid glands are clearly identifiable under xenon light as fluorescent red spots next to the thyroid glands.

**Fig 3:**
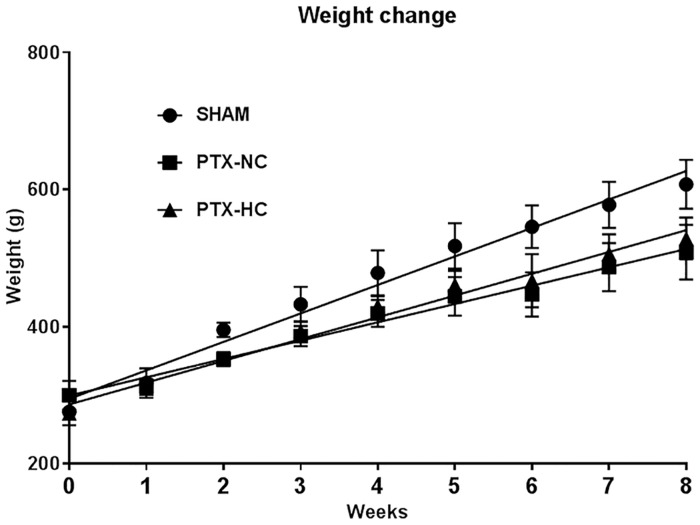
Weight changes of sham-operated and parathyroidectomized rats. All groups showed significant weight gain along the time course. (ANOVA, p = 0.000) The weight changes among the three groups were also significantly different. (ANOVA, p < 0.001) Sham-operated rats showed the greatest weight gain. Among the parathyroidectomized groups, weight gain was greatest in the parathyroidectomized animals fed a diet containing 2% calcium.

Parathyroid glands were easily identified and removed under the xenon light source (Fig 2). All five animals in the PTX-FC group were euthanized within two to four postoperative days when they showed signs of hypocalcemia (uncontrolled muscle spasm and contraction). The remaining animals survived to the end of the study (8 weeks). The serum level of PTH on postoperative 7 day was 88 ± 46 pg/mL (ranges: 37–126pg/mL) in the SHAM group. However, compared to the SHAM group, PTH in the PTX groups was decreased significantly to “non-detectable” levels. There were also remarkable differences in weight gain. The weight of SHAM group animals was 599 ± 38 g at 8 weeks. All surviving PTX group rats weighed less than the SHAM group, and the PTX-HC group weighed more than the PTX-NC group at 8 weeks (527 ± 45 and 465 ± 35 g, respectively). These results were statistically significant (Fig 3). The amount of food intake was not significantly different between the three groups.

### Serum and urine calcium and phosphorus levels

**Fig 4:**
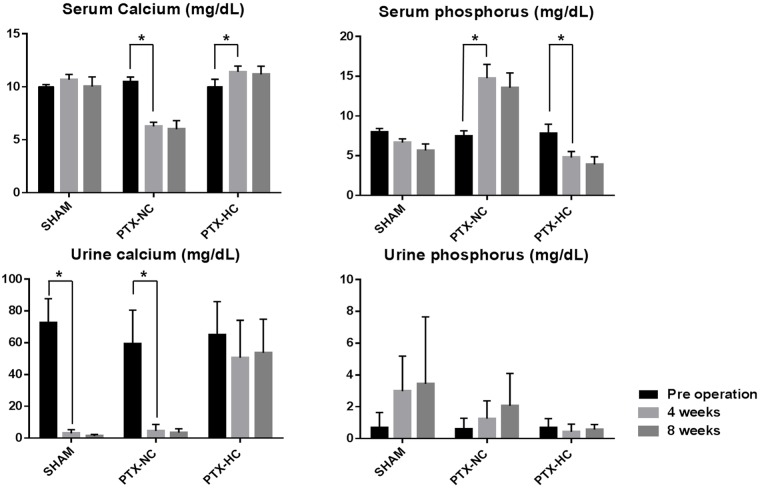
Calcium and phosphorus levels. Serum calcium levels decreased and serum phosphorus increased in the PTX-NC group after parathyroidectomy. Urine calcium levels decreased in all groups but the decrease was smallest in the PTX-HC group. Urine phosphorus increased in the SHAM and PTX-NC groups.

Compared to SHAM animals (calcium: 10.02 ± 0.91 mg/dL; phosphorous: 5.66 ± 0.81 mg/dL), serum calcium levels dropped significantly to 5.99 ± 0.81 mg/dL and serum phosphorus levels increased significantly to 13.56 ± 1.84 mg/dL in the PTX-NC group (Fig 4). In contrast, the PTX-HC group showed higher serum calcium and lower phosphorous levels than the SHAM group. There were no statistically significant differences between results at 4 and 8 weeks.

Urine calcium levels were decreased in all groups along the time course, although to a lesser extent in the PTX-HC group. Urine phosphorus levels increased in the PTX-NC and SHAM groups and decreased in the PTX-HC group (Fig 4) (Table 2). Comparing the results at 8 weeks between the groups, the PTX NC group showed higher urine calcium levels and lower urine phosphorus levels than the SHAM group. The data were provided as (S3 Table).

**Table 2:** Serum and urine calcium and phosphorus levels on postoperative 8 weeks. Data are expressed as the mean ± standard deviation.

	SHAM	PTX-NC	PTC-HC	*P*-value
Serum calcium(mg/dL)	10.02 ± 0.89	5.99 ± 0.78^a^^b^	11.17 ± 0.68^a^^b^	0.000*
Serum phosphorus(mg/dL)	5.66 ± 0.78	13.56 ± 1.76^a^^b^	3.89 ± 0.83^b^	0.000*
Urine calcium(mg/dL)	1.31 ± 0.82	3.45 ± 2.61^b^	53.68 ± 15.37^a^^b^	0.000*
Urine phosphorus(mg/dL)	3.44 ± 4.21	2.06 ± 1.86^b^	0.558 ± 0.25^b^	0.041*

*p-value < 0.05 in Kruskal-Wallis test among the three groups.

^a^Statistically significant difference between PTX groups and SHAM group by Mann-Whitney test (*p*-value < 0.05).

^b^Statistically significant difference between PTX-NC and PTX-HC groups by Mann-Whitney test (*p*-value < 0.05).

### Kidney toxicity

**Fig 5:**
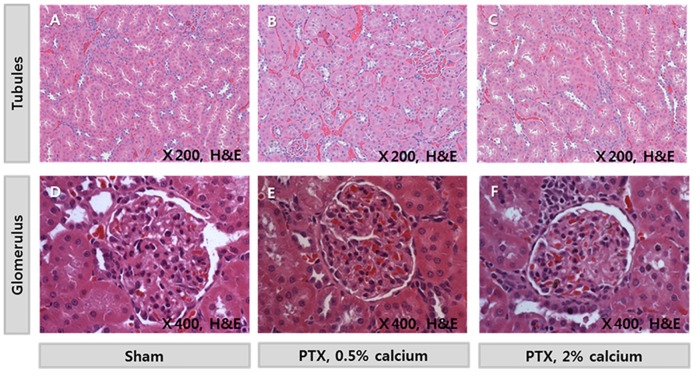
Histology of kidney glomeruli and tubules. Glomeruli and tubules exhibited normal shapes and sizes. There were no signs of tubular atrophy, interstitial fibrosis, or interstitial inflammation. Calcium deposits were not observed on microscopic examination. Tissue sections were stained with hematoxylin and eosin.

Markers of renal damage revealed that none of the diets resulted in kidney dysfunction in PTX rats. Serum BUN and creatinine levels were unchanged after 2 months of feeding each diet (Table 3). Histological evaluation demonstrated normal tubular and glomerular structures of the kidneys. There were no signs of tubular atrophy, interstitial fibrosis, interstitial inflammation, or tubular injury. Calcium phosphate deposits were not observed on tubules or vessels (Table 3 and Fig 5).

**Table 3:** Serum levels of blood urea nitrogen/ creatinine and histopathological grading of kidney at postoperative 8 weeks. Data are expressed as the means ± standard deviation. Histopathological grading is marked as 0 = normal histology, 1 = < 25%, 2 = > 25%, but <50%, 3 = > 50%, but <75%, 4 = >75%. Abbreviations: BUN, Blood urea nitrogen.

	SHAM	PTX-NC	PTC-HC	*P*-value
BUN (mg/dL)	16.73 ± 1.97	15.97 ± 2.19	18.04 ± 3.09	0.080
Creatinine (mg/dL)	0.52 ± 0.09	0.61 ± 0.05	0.60 ± 0.07	0.054
Histopathological grading	0	0	0	
Tubular necrosis	0	0	0	
Tubular dilatation	0	0	0	
Glomerular alteration	0	0	0	
Interstitial inflammation	0	0	0	
Calcium phosphate deposit	0	0	0	

### Bone turnover markers

**Fig 6:**
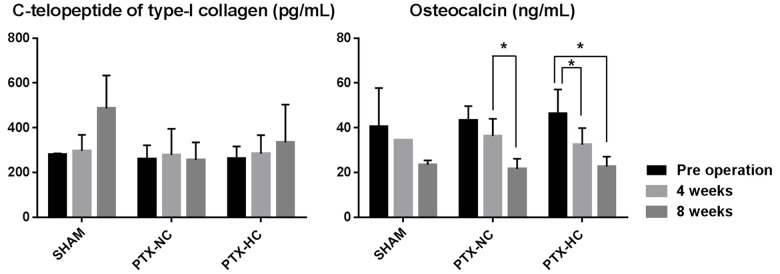
Bone turnover markers. C-Telopeptide of type I collagen levels increased in the SHAM group but not the PTX groups. Osteocalcin levels decreased in all three groups.

CTX-1 levels, an indicator of bone resorption activity, were significantly higher in SHAM than PTX animals. The CTX-1 levels of the SHAM group increased during the observational periods while the CTX-1 levels of the PTX groups were unchanged (Fig 6). Serum osteocalcin levels decreased in all three groups with time. However, there was no difference between the groups (Table 4).

**Table 4:** Bone turnover markers. Data are expressed as the means ± standard deviation.

	SHAM	PTX-NC	PTC-HC	*P*-value
CTX-1 (pg/mL)				
Pre	280.27 ± 4.45	272.25 ± 46.97	262.69 ± 54.00	
8 weeks	487.06 ± 159.97	256.56 ± 68.95^a^	304.14 ± 115.88^a^	0.020*
Osteocalcin (ng/mL)				
Pre	40.45 ± 12.35	43.31 ± 6.67	46.20 ± 9.84	
8 weeks	23.46 ± 1.97	21.67 ± 3.97	22.68 ± 4.20	0.552

*p-value < 0.05 in Kruskal-Wallis test among the three groups.

^a^Statistically significant difference between PTX groups and SHAM group by Mann-Whitney test (*p*-value < 0.05).

Abbreviations: CTX-1, C-telopeptide of type-I collagen.

### μCT results

**Fig 7:**
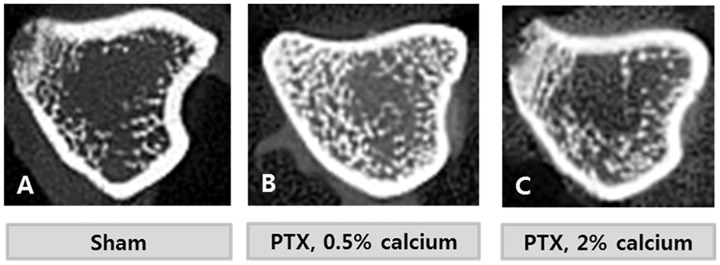
Microcomputerized tomographic images of tibia. Axial images of scans demonstrated that the trabecular bones, that display as white lines, were denser in the PTX groups than SHAM group.

Bone volume was markedly higher in the PTX groups than the SHAM group. However, there was no statistically significant difference between the PTX groups, although the PTX-NC group tended to have a higher bone volume fraction than the PTX-HC group (Table 5). Trabecular thickness, number, and sparseness did not show significant differences between the groups. Conn. D was not different among the three groups. μCT coronal images are displayed in Fig 7.

**Table 5:** Bone histomorphometry in sham-operated and parathyroidectomized groups. Data are expressed as the means ± standard deviation.

	SHAM	PTX-NC	PTC-HC	*P*-value
BV/TV (%)	17.791 ± 5.741	46.013 ± 22.29^a^	32.068 ± 6.644	0.048*
Tb.Th (mm)	0.840 ± 0.314	1.292 ± 0.577	0.821 ± 0.178	0.430
Tb.N (mm^-1^)	0.232 ± 0.097	0.349 ± 0.044	0.392 ± 0.009	0.430
Tb.Sp (mm)	2.314 ± 1.414	1.918 ± 0.647	2.509 ± 0.221	0.240
Conn.D (/ mm^3^)	0.738 ± 0.405	0.725 ± 0.498	0.862 ± 0.014	0.080

*p-value < 0.05 in Kruskal-Wallis test among the three groups.

^a^Statistically significant difference between PTX groups and SHAM group by Mann-Whitney test (*p*-value < 0.05).

Abbreviations: BV/TV, Bone volume fraction; Tb.Th, Trabecular thickness; Tb.Sp, Trabecular separation; Tb.N, Trabecular number; Conn.D, Connectivity density.

## Discussion

Disease-specific animal models are invaluable for evaluating newly developed therapeutic modalities. Ideal animal models should be reproducible, cost-effective, and accurately represent human physiology and anatomy of the disease to make study results plausible. [14] Methods for creating such animal models include surgery, radiation exposure, diet control, genetic knockout, and drug administration. In addition to the creation methodology, animals should be acclimated and maintained in appropriate and controlled conditions to represent the pathophysiology of the disease.

Animal models of hypoparathyoidism have been reported. [6–10] Most of these models involved removing the parathyroid gland surgically using microscopic excision and bipolar cauterization; however, postoperative PTH levels were inconsistent. [9] Because rat parathyroid glands are very small, it is difficult to distinguish these glands from the thyroid without special identification methods. Uncertain identification may lead to partial parathyroidectomy or excessive excision including the thyroid gland. Incomplete excision results in varying postoperative PTH levels. Disrupting thyroid gland tissue may cause bleeding during the procedure and even hypothyroidism when the excised amount is excessive.

We have previously decreased PTH levels successfully by performing parathyroidectomy. [4, 5, 11] Parathyroid glands were excised by using a fluorescent-identification method, and post-operatively, PTH decreased to undetectable levels. In the present study, the death of rats in the PTX-FC group, and undetectable postoperative PTH levels in all PTX group animals, demonstrated that this surgical method was highly effective and reproducibly generated an animal model of hypoparathyroidism.

In addition to an effective method for removing the parathyroid gland, maintaining the animal’s condition is necessary to conduct further studies. Considering the pathophysiology of hypoparathyroidism, the diet formula for animals, especially calcium and phosphorus content, should be modulated and controlled. Diet formulations with poorly controlled calcium concentrations could result in inconsistent serum calcium levels. Unlike our previous study that used cereal-based rodent chows [11], purified diets were used to maintain the animals in the current study. While cereal-based chow has the benefit of low cost, the nutrient components, including minerals, are variable in each supplement. The AIN-93G diet is a widely-used purified diet composed of isolated proteins, sugars, oils, and purified sources of vitamins and minerals. [12] By maintaining a strict balance of the calcium-phosphorus ratio, this formula decreased calcium deposits on the kidney relative to the former diets. [13]

Evaluating the calcium content of the AIN-93G diet is necessary to maintain the hypoparathyroidism animal model. Excessively high calcium concentrations in the diet formula can disturb the pathophysiology of hypothyroidism, and low calcium concentrations may cause death of the animals. The ideal calcium concentration is one that allows the animals to survive while making them present the pathophysiology of hypoparathyroidism throughout the observational period. A CFD was used to maximize the effect of low PTH levels on our animal model, while AIN-93G diets with normal and high calcium levels were used as treatment formula to enable animals to survive after the PTX procedure. Although vitamin D analogues are used to treat hypoparathyroidism in humans, only calcium was added in this study because rodents can obtain vitamin D by fur grooming. [15–17]

The CFD dramatically decreased serum calcium levels of animals when it was combined with PTX, and combined group (PTX-FC) rats did not survive beyond four days. Tetany was observed in all PTX-FC animals. Death of the rats demonstrated that this formula was incompatible for use with PTX as an animal model. Rats fed the AIN-93G diet (PTX-NC) demonstrated promising results. Unlike the PTX-FC group, these animals survived the entire observation period without further treatment. This showed that the calcium concentration of AIN-93G diets is sufficient to keep the animals alive.

The pathophysiology of hypothyroidism was not distorted by the AIN-93G diet. Compared to the SHAM group, the PTX-NC group showed lower serum and higher urine calcium levels. These levels demonstrated decreased calcium resorption in kidney, which is in close agreement with human hypoparathyroidism. [1] Phosphorous levels were higher in serum and lower in urine in the PTX-NC group than the SHAM group. These changes were consequences of the decreased calcium resorption that led to decreased phosphorous excretion by the kidney. When comparing the PTX-NC and PTX-HC groups, the effect of dietary calcium levels became more obvious. When the calcium concentration of the diet was increased (PTX-HC group), serum calcium levels were higher than the SHAM group despite undetectably low PTH levels. Serum phosphorus levels were lower in the PTX-HC group than the SHAM group. Overall, the calcium-phosphorus balance was disturbed by the high calcium diet indicating that supplementation with excess calcium was inappropriate in this animal model to evaluate the feasibility of treatment modalities.

Because PTH plays a role in bone resorption, hypoparathyroidism patients exhibit higher bone volume than individuals with a normal PTH level. Bone histomorphology of the PTX-NC group was representative of hypoparathyroidism. Bone volume and trabecular thickness were higher than in the SHAM group based on μCT analysis. When dietary calcium supplementation was increased (PTX-HC group), the changes of bone morphology were less dramatic than in the group fed the normal AIN-93G diet. Bone turnover markers demonstrated similar results to the μCT analysis. CTX-1 levels which represented the bone resorption activity were lower in the PTX groups than the SHAM group. However, as a function of CTX-1 level increased in SHAM group while there were no significant changes in PTX groups. These results could be explained by the age of the Sprague Dawley rats used in this study. The animals were 2 to 4 months old during the observational period. This age equates to human puberty with growth spurt. [18] CTX-1 levels increased during the growth spurts while osteocalcin levels are constant. [19]

PTH stimulates the kidney to excrete calcium and reabsorb phosphorus. Urine calcium levels were elevated when high calcium diets were fed to hypoparathyroidism animals. Increased urine calcium levels could cause calcium deposits on kidney tissue and tubular injury. However, in this current study, calcium deposition and kidney injury were not observed on histologic evaluation. A longer observational period would be required for precise effect of high calcium diet on kidney.

The normal AIN-93G diet is less expensive ($36/kg) than the two special-order diets ($54/kg). When fed a CFD, all animals died shortly after parathyroidectomy. This early death caused an unnecessary waste of money and animals. Because all animals in this study survived when they were fed the normal AIN-93G diet, calcium addition was unnecessary.

In this study, only male rats were used to avoid the sex-hormonal effect on calcium-related hormones and reduce the sample bias. [20] However, the long-term observational animal experiment with female rat could be considered to evaluate the feasibility of the hypoparathyroidism animal model in future study.

We suggest that this hypoparathyroidism animal model produced by parathyroidectomy is highly reproducible when using the fluorescent-identification method and the animals are fed the standard purified AIN-93G diet. The results also demonstrate that this animal model exhibits pathophysiology similar to human primary hypoparathyroidism at a reasonable cost.

## Conclusions

The fluorescent-identification method that uses intraperitoneal 5-ALA injection and ultraviolet light examination is a novel surgical technique to yield fully parathyroidectomized rats. Furthermore, feeding these rats a purified AIN-93G diet containing 0.5% calcium is sufficient for maintaining these animals. This hypoparathyroidism animal model is reproducible, cost-effective, and exhibits pathophysiology similar to human hypoparathyroidism.

## Supporting Information

S1 Table Formulation of the AIN-93G diet. (PDF)

S2 Table Mineral mix of the AIN-93G diet. (PDF)

S3 Table Laboratory results of the animal experiments. (DOCX)
