# Supplementary material for: Standardization of A Physiologic Hypoparathyroidism Animal Model
Source: PLoS One. 2016 Oct 3;11(10):e0163911. doi: 10.1371/journal.pone.0163911 (PMC5047647; doi:10.1371/journal.pone.0163911)
Supplement: S1 Table — (PDF) [file pone.0163911.s001.pdf]

Supplement 1. Formulation of the AIN-93G diet.

Ingredient	g/ kg diet
Corn starch	397
Casein	200
Maltodextrin	132
Sucrose	100
Soybean oil	70
Cellulose	50
Mineral mix	35
Vitamin mix	10
L-Cystine	3
Choline bitartrate	2.5
t-Butylhydroquinone	0.014
