# Supplementary material for: Standardization of A Physiologic Hypoparathyroidism Animal Model
Source: PLoS One. 2016 Oct 3;11(10):e0163911. doi: 10.1371/journal.pone.0163911 (PMC5047647; doi:10.1371/journal.pone.0163911)
Supplement: S2 Table — (PDF) [file pone.0163911.s002.pdf]

Supplement 2. Mineral mix of the AIN-93G diet.

Ingredient	g/ kg diet
Calcium carbonate	357
Potassium phosphate	196
Potassium citrate	70.78
Potassium sulfate	46.6
Magnesium oxide	24
Sodium chloride	74
Ferric citrate	6.06
Zinc carbonate	1.65
Sodium silicate	1.45
Manganous carbonate	0.63
Cupric carbonate	0.3
Chromium potassium sulfate	0.275
Boric acid	0.0815
Sodium fluoride	0.0635
Nickel carbonate	0.0318
Lithium chloride	0.0174
Sodium selenate	0.01025
Potassium iodate	0.01
Ammonium molybdate	0.00795
Ammonium vanadate	0.0066
Powdered sucrose	221
