# Supplementary material for: Standardization of A Physiologic Hypoparathyroidism Animal Model
Source: PLoS One. 2016 Oct 3;11(10):e0163911. doi: 10.1371/journal.pone.0163911 (PMC5047647; doi:10.1371/journal.pone.0163911)
Supplement: S3 Table — (DOCX) [file pone.0163911.s003.docx]

Supplement 3. Laboratory results of the animal experiments.
